# Dual solution framework for mixed convection flow of Maxwell nanofluid instigated by exponentially shrinking surface with thermal radiation

**DOI:** 10.1038/s41598-021-95548-9

**Published:** 2021-08-05

**Authors:** Qiu-Hong Shi, Bilal Ahmed, Sohail Ahmad, Sami Ullah Khan, Kiran Sultan, M. Nauman Bashir, M. Ijaz Khan, Nehad Ali Shah, Jae Dong Chung

**Affiliations:** 1 Department of Mathematics, Huzhou University, Huzhou, 313000 People’s Republic of China; 2 Department of Mathematics and Statistics, The University of Lahore, Sargodha Campus, Sargodha, 40100 Pakistan; 3 Department of Mathematics, COMSATS University Islamabad, Sahiwal, 57000 Pakistan; 4 Department of Mathematics and Statistics, Riphah International University I-14, Islamabad, 44000 Pakistan; 5 Nonlinear Analysis and Applied Mathematics (NAAM) Research Group, Department of Mathematics, Faculty of Science, King Abdulaziz University, P.O. Box 80257, Jeddah, 21589 Saudi Arabia; 6 Department of Mechanical Engineering, Sejong University, Seoul, 05006 Korea; 7 Department of Mathematics, Lahore Leads University, Lahore, Pakistan

**Keywords:** Engineering, Mathematics and computing

## Abstract

This paper presents the analysis of transfer of heat and mass characteristics in boundary layer flow of incompressible magnetohydrodynamic Maxwell nanofluid with thermal radiation effects confined by exponentially shrinking geometry. The effects of Brownian motion and thermophoresis are incorporated using Buongiorno model. The partial differential equations of the governing model are converted in non-dimensional track which are numerically inspected with proper appliances of Runge–Kutta fourth order scheme.The significant effects of heat and mass fluxes on the temperature and nanoparticles volume fractions are investigated. By the increases in Lewis number between $1.0$ to $2.0$, the decrease in nanoparticle volume fraction and temperature is noted. With the change in the Prandtl constant that varies between $0.7$ to $1.5$, the nanoparticles volume fraction and temperature are dwindled. Nanoparticles volume fraction and temperature distribution increase is noted with applications of radiation constant. With consequent variation of thermophoresis parameter between $0.1$ to $0.8$, nanoparticles volume fraction and temperature distribution increases. It is also noted that the increase in thermophoresis parameter and Brownian parameter from $0.1$ to $0.8$, nanoparticles volume fraction decreases while temperature distribution increases.

## Introduction

Study of nano-materials configured by shrinking/stretching sheet with different parameters is observed rapidly in past few decades. The interest of scholars and scientists to study the field of nanofluid is increased due to vast applications of nanofluid in the industrial and contemporary technology. For the first time the boundary layer flow over plane stretching sheet was analysed by Crane^1^, the transfer of heat and mass for different conditions was than included as an extension in the work of Crane by Gupta et al.^2^, Chen and Char^3^ and Dutta and co-investigators^4^. In the investigations of these scholars mentioned above the occurrence of flow of fluid was caused due to stretching velocity produced by shrinking sheet. As industries and metallurgy are the need of an hour, the magneto hydrodynamics and transfer of heat in boundary layer flow is the focus of study for different researchers. These factors are also studied for different applications in engineering fields such as extraction of geothermal energy, growing of crystals, planting the power houses, study of plasma, production of papers and generators with MHD phenomena. For the history of study of stretching sheet the first name that comes ahead is Sakiadis^5–7^. He presented the flow of fluid produced due to stretching surface. The flow due to nonlinear and linear stretching surface became the focus that was investigated by Zheng^8^, Zheng et al.^9^, Zheng et al.^10^. The exponentially stretching sheet became the focus of study for the different researchers. Sajid and Hayat^11^, Magyari and Keller^12^ analysed the thermal radiation over exponentially stretching surface, which opened a new gateway for different researcher. Mukhophadhyay^13,14^ investigated the thermally stratified and porous medium in an exponentially stretching surface. This type of flow was then analysed for different type of fluids by different researchers^15,16^. The effects as viscous dissipation, double diffusion and mixed convection for such flow over stretching surface were then analysed by Patil et al.^17^. The references from^18–26^ reflect the study of transfer and flow of heat in a viscid and non-viscous fluid for exponentially stretching sheet. The distinct outcomes of non-Newtonian materials presented the platform to researches recently. This is due to the vast usage of non-Newtonian fluids in the industrial areas. Types of non-Newtonian fluid are categorized in integral, differential and rate types. Maxwell fluids are rate type non-Newtonian, non-viscid fluid. The exact solutions for flow of Maxwell fluid is analysed by Fetecau^27^. The Maxwell fluid mechanism in porous space has beenanalyzed by Wang and Hayat^28^. The Maxwell fluid flow in unsteady space was directed by Fetecau et al.^29^. A 2-D MHD Maxwell fluid flow was analysed by Hayat et al.^30^. As predicted from above study that the flow simulations against mass/heat transportation over permeable medium or sheet has gained attraction of different investigators with fact of its vast industrial applications and in technology. To increase the rate of transfer of heat on surface the porous material is mainly considered. Nanotechnology became of eyes of researchers in few past years. It has become a new exciting frontier in the fields of technology. It is because the applications exerted from the nanofluids. Nanofluid is a fluid containing a base fluid with Nano size particles that helps to increases the thermal conductivity of various solids and liquids. Nano fluids shows great thermo physical properties such as thermal diffusion, thermal conductivity, it hence the rate of transfer of heat, reduces viscosity and much more. But the key feature of the nanofluid is superior thermal conductivity, which reduces many problems. Nano-fluids offer us quite efficient and greener solution to our current technological problems. Nano-fluid is the next possible replacement for enhancement and effectiveness of technology. The outcomes for thermodiffusion and Brownian aspects in nanofluids with the heat and mass fluxes were represented by Mukhphadyay and Ghosh^31^. Bachok et al.^32^ studied the transfer of heat of nanofluid over porous stretching and shrinking sheet and represented the dual solutions for them. MHD stagnation point unsteady flow and transfer of heat of nanofluids on shrinking and stretching sheet were analysed by Khalili et al.^33^. Sreedevi et al.^34^ presented the analysis of single and multi-wall nano tubes over vertical cone under the inducement of magnetic field. In another investigation Sreedevi et al.^35^ discussed the hat and mass transfer of the flow of nano fluid over a cone saturated in porous medium to present the effects pf suction and injection phenomenon. Sreedevi and Reddy^36^ studied the flow of hydromagnetic nano-Maxwell fluid sandwiched between two rotating stretchable disks. Under the assumptions of boundary layer approximation the flow of nano fluid over a cone with chemical reaction was studied by Reddy et al.^37^. Recently, many investigation^38–42^ were made in order to resent the analysis of nanofluid in a different physical situations.

Our present work is about the study of the transfer of heat and flow of Maxwell nanofluids with heat and mass fluxes over porous exponentially shrinking sheet with MHD and thermal radiation effects. Going deep in the literature of research we found out that Maxwell non-Newtonian fluids are not discussed and analysed before on the shrinking sheet.It is difficult to handle the solutions of Maxwell non-Newtonian fluids with shrinking effect of sheet. This is why it is not analysed till now. The purpose of the present study is to provide mathematical modelling, numerical simulation and analysis of the existence of the dual solution of the flow of Maxwell nano-fluid over a shrinking sheet under the inducement of magnetic field.

## Problem formulation

**Figure 1:**
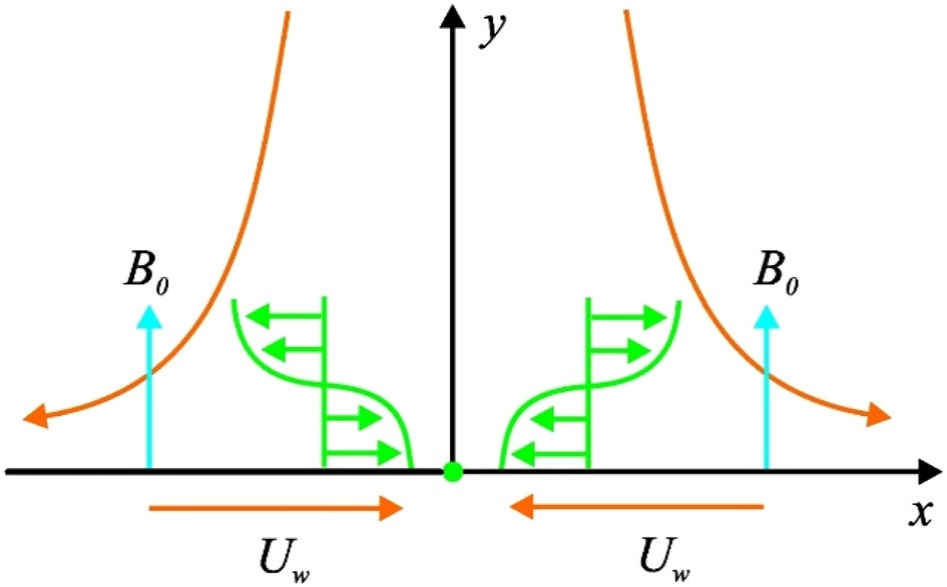
Schematic flow diagram.

Consider a two-dimensional, two-directional flow of a Maxwell nanofluid which is electrically conducting amassed incompressible over an exponentially shrinking sheet. The magnetic field consequences are accounted perpendicular to the flow zone as shown in Fig. 1. The assumptions of low magnetic Reynolds number lead to abandon of induced magnetic features. The flow is intended in *x*-direction while $y$-axis is considered normally.

The steady boundary layer incompressible viscous MHD Maxwell nanofluid flow is studied over exponentially shrinking sheet with mass and heat fluxes. The assumptions under considerations lead to following flow equations:

1 $$\frac{\partial u}{\partial x}+\frac{\partial v}{\partial y}=0,$$

2 $$u\frac{\partial u}{\partial x}+v\frac{\partial u}{\partial y}=\upsilon \frac{{\partial }^{2}u}{{\partial y}^{2}}-{\Lambda }_{1}\left[{u}^{2}\frac{{\partial }^{2}u}{{\partial x}^{2}}+{v}^{2}\frac{{\partial }^{2}u}{{\partial y}^{2}}+2uv\frac{{\partial }^{2}u}{\partial x\partial y}\right]-\frac{\sigma {B}_{0}^{2}}{{\rho }_{f}}\left[{\Lambda }_{1}v\frac{\partial u}{\partial y}+u\right],$$

3 $$u\frac{\partial T}{\partial x}+v\frac{\partial T}{\partial y}=\alpha \frac{{\partial }^{2}T}{{\partial y}^{2}}+\frac{{\left(\rho c\right)}_{P}}{{\left(\rho c\right)}_{f}}\left[{D}_{B}\frac{\partial N}{\partial y}\frac{\partial T}{\partial y}+\frac{{D}_{T}}{{T}_{\infty }}{\left(\frac{\partial T}{\partial Y}\right)}^{2}\right]-\frac{1}{\rho {c}_{p}}\frac{\partial {q}_{r}}{\partial y},$$

4 $$u\frac{\partial N}{\partial x}+v\frac{\partial N}{\partial y}={D}_{B}\frac{{\partial }^{2}N}{{\partial y}^{2}}+\frac{{D}_{T}}{{T}_{\infty }}\frac{{\partial }^{2}T}{{\partial y}^{2}},$$

where $u$ and $v$ are velocity components of our considered nano-Maxwell fluid flow along $x$ and $y$ directions respectively, $\upsilon$ symbolized the kinematic viscosity, ${\Lambda }_{1}$ is the relaxation time, $\sigma$ is the factor showing that our fluid is electrically conducting, ${D}_{B}$ Browniandiffusion,$B$ exhibit variable magnetic field,$N$ nanoparticles volume fraction,${B}_{O}$ is a constant, collectively $\frac{\sigma {B}_{0}^{2}}{{\rho }_{f}}\left[\lambda v\frac{\partial u}{\partial y}+u\right]$ is the Lorentz force,${\rho }_{f}$ base fluid density, $\alpha$ exhibit thermal conductivity, $({\rho c)}_{p}$ is effective heat capacity of nanoparticles, $({\rho c)}_{f}$ is nanoparticle volume fraction, $T$ exhibits temperature, ${T}_{\infty }$ is a constant free stream temperature and ${D}_{T}$ is thermophoretic diffusion.

For the radiation heat flux, ${q}_{r}$ is used in Eq. (3),${q}_{r}$ via Rosseland approximation is also written as ${q}_{r}=-\frac{4{\sigma }^{*}}{3{k}^{*}}\frac{\partial {T}^{4}}{\partial y}$. Here, ${\sigma }^{*}$ is a Stephen Boltzmann constant and ${k}^{*}$ is mean absorption coefficient. Within the viscous fluid flow the less temperature gradient is assumed which expresses ${T}^{4}$ as a linear function of temperature. Using Taylor’s series ${T}^{4}$ is expanded about a free stream temperature ${T}_{\infty }$ as shown below.

5 $${T}^{4}={{T}_{\infty }}^{4}+4{{T}_{\infty }}^{3}\left(T-{T}_{\infty }\right)+6{{T}_{\infty }}^{2}{\left(T-{T}_{\infty }\right)}^{2}+\dots $$

6 $$\begin{array}{cc}\therefore & \frac{\partial {q}_{r}}{\partial y}=-\frac{16{\sigma }^{*}}{3{k}^{*}}{{T}_{\infty }}^{3}\frac{{\partial }^{2}T}{\partial {y}^{2}}.\end{array}$$

The subjected boundary conditions are

7 $$\begin{array}{cc}\begin{array}{ccc}u={U}_{w},& v=-V\left(x\right),& T={T}_{\infty }+{T}_{0}{ e}^{x/2L},\end{array}& \begin{array}{ccc}N={N}_{\infty }+{N}_{0}{ e}^{x/2L}& \mathrm{at}& y=0\end{array}\end{array}$$

8 $$\begin{array}{cc}\begin{array}{ccc}& u\to 0,& T\to {T}_{\infty },\end{array}& \begin{array}{ccc}N\to {N}_{\infty }& \mathrm{as}& y\to \infty .\end{array}\end{array}$$

Here ${U}_{w}={-U}_{0}{e}^{x/L}$ is shrinking velocity,${U}_{O}$ is a reference velocity,$V\left(x\right)=-{v}_{o}{e}^{x/2L}$ is a shrinking velocity at the wall, where ${v}_{O}$ is a constant.

Let us introduce the similarity transformation

9 $$\begin{array}{ccc}\psi =\sqrt{2\nu L{U}_{O}}f\left(\eta \right){e}^\frac{x}{2L}, & \eta =y\sqrt{\frac{{U}_{o}}{2\nu L}}{e}^\frac{x}{2L},& N={N}_{\infty }+{N}_{o}{e}^\frac{x}{2L}\phi \left(\eta \right),\\ v=-\sqrt{\frac{{U}_{o}\nu }{2L}}{e}^\frac{x}{2L}\left(\eta {f}^{\mathrm{^{\prime}}}\left(\eta \right)+f\left(\eta \right)\right),& u={{U}_{O}f}^{\mathrm{^{\prime}}}{\left(\eta \right)e}^\frac{x}{L},& T={T}_{\infty }+{T}_{o}{e}^\frac{x}{2L}\theta \left(\eta \right),\end{array}$$

where $\psi$ and $\eta$ are being stream function and similarity variable, respectively. After using Eq. (8) in Eqs. (1)-(4), we get

10 $${f}^{\mathrm{^{\prime}}\mathrm{^{\prime}}\mathrm{^{\prime}}}+f{f}^{\mathrm{^{\prime}}\mathrm{^{\prime}}}-2{{f}^{\mathrm{^{\prime}}}}^{2}-2{M}^{2}{f}^{\mathrm{^{\prime}}}+\lambda \left(\begin{array}{c}3f{f}^{\mathrm{^{\prime}}}{f}^{\mathrm{^{\prime}}\mathrm{^{\prime}}}+\frac{\eta }{2}{{f}^{\mathrm{^{\prime}}}}^{2}{f}^{\mathrm{^{\prime}}\mathrm{^{\prime}}}\\ -2{{f}^{\mathrm{^{\prime}}}}^{3}-\frac{1}{2}{{f}^{2}f}^{\mathrm{^{\prime}}\mathrm{^{\prime}}\mathrm{^{\prime}}}\end{array}\right) +{M}^{2}\lambda \left(\eta {f}^{\mathrm{^{\prime}}}{f}^{\mathrm{^{\prime}}\mathrm{^{\prime}}}+f{f}^{\mathrm{^{\prime}}\mathrm{^{\prime}}}\right)=0,$$

11 $$\left(1+\frac{4}{3}Rd\right){\theta }^{\mathrm{^{\prime}}\mathrm{^{\prime}}}+\mathrm{Pr}\left(f{\theta }^{\mathrm{^{\prime}}}-{f}^{\mathrm{^{\prime}}}\theta +{N}_{b}{\phi }^{\mathrm{^{\prime}}}{\theta }^{\mathrm{^{\prime}}}+{N}_{t}{{\theta }^{\mathrm{^{\prime}}}}^{2}\right)=0,$$

12 $${\phi }^{\mathrm{^{\prime}}\mathrm{^{\prime}}}+PrLe\left(f{\phi }^{\mathrm{^{\prime}}}-{f}^{\mathrm{^{\prime}}}\phi \right)+\frac{{N}_{t}}{{N}_{b}}{\theta }^{\mathrm{^{\prime}}\mathrm{^{\prime}}}=0.$$

Here Eq. (1) is satisfied identically and the parameters $\mathrm{Pr}, Le, M, {N}_{b}, {N}_{t}, \lambda , Rd, Sc, \gamma$ involved in the governing equations are Prandtl number, Lewis number, Hartmann number, Brownian motion and thermophoresis parameters, Deborah number, Radiation parameter, Schmidt number, Biot number respectively defined as followed

13 $$\begin{array}{ccc}Pr=\frac{\nu }{\mathrm{\alpha }},& \lambda =\frac{{\Lambda }_{1}{U}_{o}{e}^\frac{x}{L}}{L}& {N}_{b}=\frac{{\left(\rho c\right)}_{p}}{{\left(\rho c\right)}_{f}}{N}_{o}\frac{{\mathrm{D}}_{B}}{\nu }{e}^\frac{x}{2L},\\ Le=\frac{\mathrm{\alpha }}{{D}_{B}},& Rd=\frac{4{\sigma }^{*}{{T}_{\infty }}^{3}}{\rho {c}_{p}{k}_{1\mathrm{\alpha }}}& {N}_{t}= \frac{{\left(\rho c\right)}_{p}}{{\left(\rho c\right)}_{f}}\frac{{D}_{T}}{{T}_{\infty }}\frac{1}{\nu }{T}_{o}{e}^\frac{x}{2L},\end{array}$$

and ${M}^{2}=\sigma L{{B}_{o}}^{2}/\rho {U}_{O}{e}^\frac{x}{L}$ is magnetic parameter. The developed boundary conditions are:

14 $$\begin{array}{ccc}\begin{array}{cc}\begin{array}{cc}f\left(\eta \right)=S,& {f}^{\mathrm{^{\prime}}}\left(\eta \right)=-1,\end{array}& \begin{array}{cc}\theta \left(\eta \right)=1,& \phi (\eta )=1,\end{array}\end{array}& \mathrm{at}& \eta =0\end{array}$$

15 $$\begin{array}{ccc}\begin{array}{ccc}{f}^{\mathrm{^{\prime}}}\left(\eta \right)\to 0,& \theta \left(\eta \right)\to 0,& \phi (\eta )\to 0.\end{array}& \mathrm{as}& \eta \to \infty \end{array}$$

Here $S=-{v}_{o}/\sqrt{\nu {U}_{w}/2L}$ is suction and injection parameter. When $S<0$, it indicates mass injection and when $S>0$, it indicates mass suction. The local Sherwood, wall shear forceand local Nusselt numbers are communicated below to indicate heat and mass transfer

16 $$\begin{array}{ccc}Sh=\frac{{xj}_{i}}{{D}_{B}({N}_{\infty }-{N}_{w})},& {C}_{f}=\frac{{\tau }_{i}}{\rho {{U}_{w}}^{2}},& Nu=\end{array}\frac{{xq}_{i}}{{K}_{B}({T}_{\infty }-{T}_{w})}.$$

Here ${j}_{i}$, ${\tau }_{i}$, ${q}_{i}$ are mass, momentum and heat fluxes from the surface. They are defined as

17 $$\begin{array}{ccc}{j}_{i}=-{D}_{B}{\left[\frac{\partial N}{\partial y}\right]}_{y=0},& {\tau }_{i}=\mu \left(1+\lambda \right){\left[\frac{\partial u}{\partial y}\right]}_{y=0},& {q}_{i}=-{K}_{B}\left(\frac{{q}_{r}}{\alpha \left(\rho c\right)p}+{\left[\frac{\partial T}{\partial y}\right]}_{y=0}\right).\end{array}$$

In the dimensionless form, Eq. (16) becomes

18 $$\begin{array}{ccc}\frac{{Nu}_{x}}{\sqrt{{\mathrm{Re}}_{x}}}=-\sqrt{\frac{x}{2L}} \left(1+\frac{4}{3}Rd\right){\theta }{^\prime}\left(0\right),& \sqrt{2{\mathrm{Re}}_{x}{C}_{f}}={\left(1+\lambda \right)f}^{\prime\prime}\left(0\right),& \frac{{Sh}_{x}}{\sqrt{{\mathrm{Re}}_{x}}}=-\sqrt{\frac{x}{2L}} {\phi }^{\prime}\left(0\right)\end{array}$$

where ${\mathrm{Re}}_{x}=\frac{{U}_{w}x}{\nu }$ is the local Reynolds number.

## Numerical simulation

The numerical procedure based on Runge–Kutta fourth order scheme with appliances of secant shooting scheme is employed in order to present the numerical simulations. The secant shooting approach is preferable over simple shooting procedure due to fact that simple shooting technique involves the derivative of the system and then approximates the missing condition while in the secant shooting method, missing condition can be approximated without finding the derivative of the whole system. With high range accuracy and convergence, the secant shooting scheme is the most effective approach for such types of nonlinear problems. This scheme is proceeded as:

Equations (10)–(12) are altered into first order system by adjusting $f={f}_{1}$, $\theta ={f}_{4}$ and $\phi ={f}_{6}$ and we have

19 $${{f}_{1}}{^{\prime}}={f}_{2},$$

20 $${f}_{2}^{{\prime}}={f}_{3},$$

21 $${f}_{3}^{{\prime}}=\frac{1}{1-\frac{1}{2}\lambda {{f}_{2}}^{2}}\left(\begin{array}{c}2{{f}_{2}}^{2}-{f}_{1}{f}_{3 }+2{M}^{2}{f}_{2}-\lambda \left(3{f}_{1}{f}_{2}{f}_{3}+\frac{\eta }{2}{{f}_{2}}^{2}{f}_{3}-2{{f}_{2}}^{3}\right)\\ -{M}^{2}\beta \left(\eta {f}_{2}{f}_{3}+{f}_{1}{f}_{3}\right)\end{array}\right),$$

22 $${f}_{4}^{{\prime}}={f}_{5},$$

23 $${f}_{5}^{{\prime}}=\frac{\mathrm{Pr}}{1+\frac{4}{3}Rd}\left({f}_{2}{f}_{4}-{f}_{1}{f}_{5}-{N}_{b}{f}_{7}{f}_{5}-{N}_{t}{{f}_{5}}^{2}\right),$$

24 $${f}_{6}^{\prime}={f}_{7}$$

25 $${f}_{7}^{\prime}=-PrLe\left[{f}_{1}{f}_{7}-{f}_{2}{f}_{6}\right]+\frac{{N}_{t}\mathrm{Pr}}{{N}_{b}\left(1+\frac{4}{3}Rd\right)}\left({f}_{2}{f}_{4}-{f}_{1}{f}_{5}-{N}_{b}{f}_{7}{f}_{5}-{N}_{t}{{f}_{5}}^{2}\right)$$

and boundary condition becomes

26 $$\begin{array}{cc}\begin{array}{cc}{f}_{1}\left(0\right)=S,& {f}_{2}\left(0\right)=-1,\end{array}& \begin{array}{cc}{f}_{4}\left(0\right)=1,& {f}_{6}(0)=1,\end{array}\end{array}$$

27 $$\begin{array}{ccc}{f}_{2}\left(L\right)\to 0,& {f}_{4}\left(L\right)\to 0,& {f}_{6}(L)\to 0.\end{array}$$

The increment in L make the convergence procedure more effective and appropriate. Assuming the missing initial conditions as follows

28 $$\begin{array}{ccc}{f}_{3}\left(0\right)={m}_{1},& {f}_{5}\left(0\right)={m}_{2},& {f}_{4}\left(0\right)={m}_{3}.\end{array}$$

(i) Integrating the Eqs. (19)–(25) subject to conditions given in (26) and (28) as an initial value system by providing the initial guess to ${m}_{i}$ say ${{m}_{i}}^{(0)}$ and ${{m}_{i}}^{(1)}$ where $i=1, 2, 3$.
(ii) Approximate the ${m}_{i}$ by using the secant method defined by
  29 $${{m}_{i}}^{(n+1)}={{ m}_{i}}^{(n)}-{f}_{\left(j\right)}\left(L,{{ m}_{i}}^{\left(n\right)}\right)\frac{{{ m}_{i}}^{\left(n\right)}-{{ m}_{i}}^{\left(n-1\right)}}{{f}_{\left(j\right)}\left(L,{{ m}_{i}}^{\left(n\right)}\right)-{f}_{\left(j\right)}\left(L,{{ m}_{i}}^{\left(n-1\right)}\right)}.$$
(iii) Repeat the steps (iii) and (iv) until significance convergence is achieved.
(iv) Simulations are performed with MATLAB algorithm.

## Result and discussion

**Figure 2:**
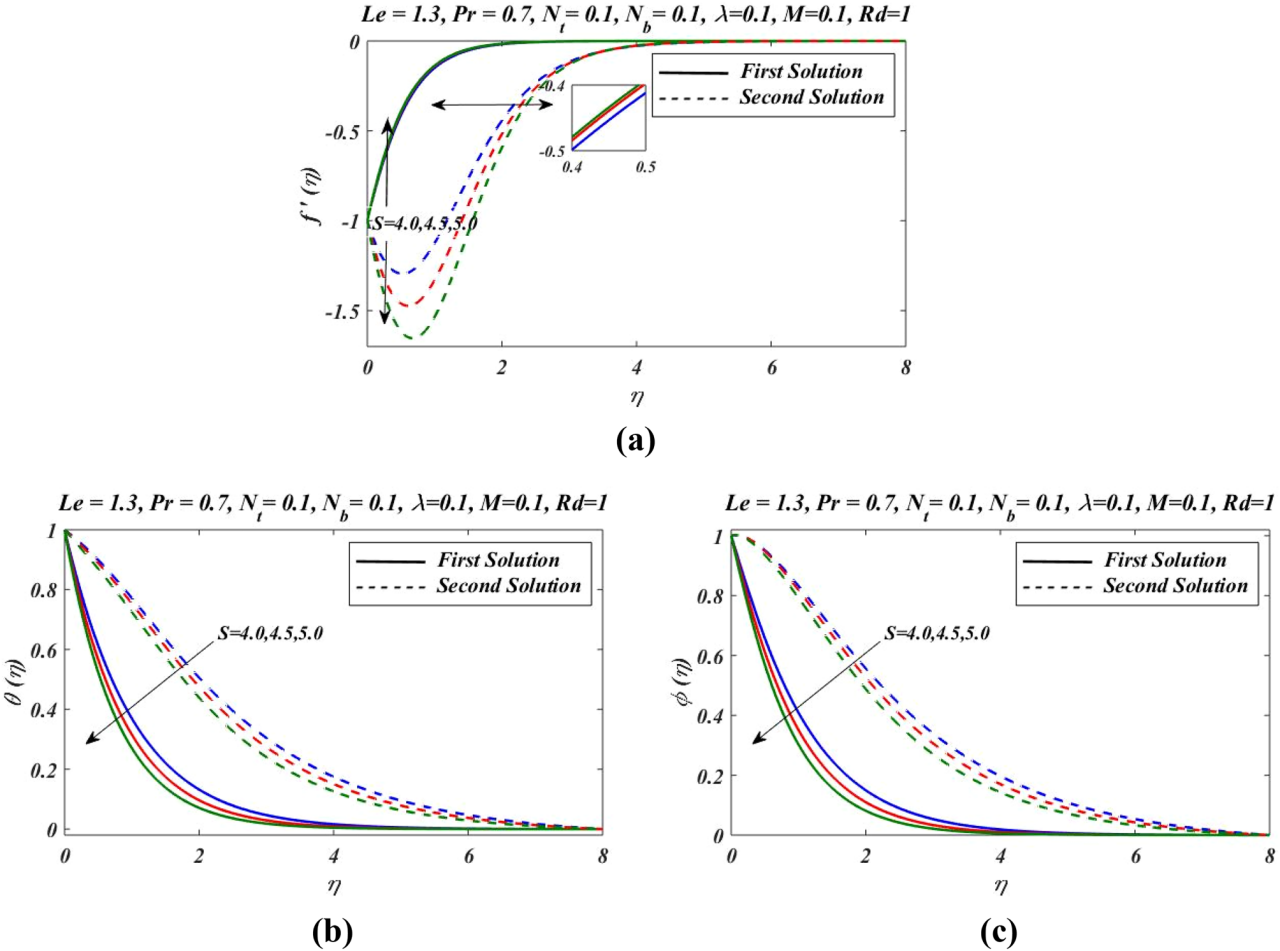
Outcomes of $S$ on (**a**) velocity, (**b**) temperature and (**c**) nanoparticles volume fraction.

**Figure 3:**
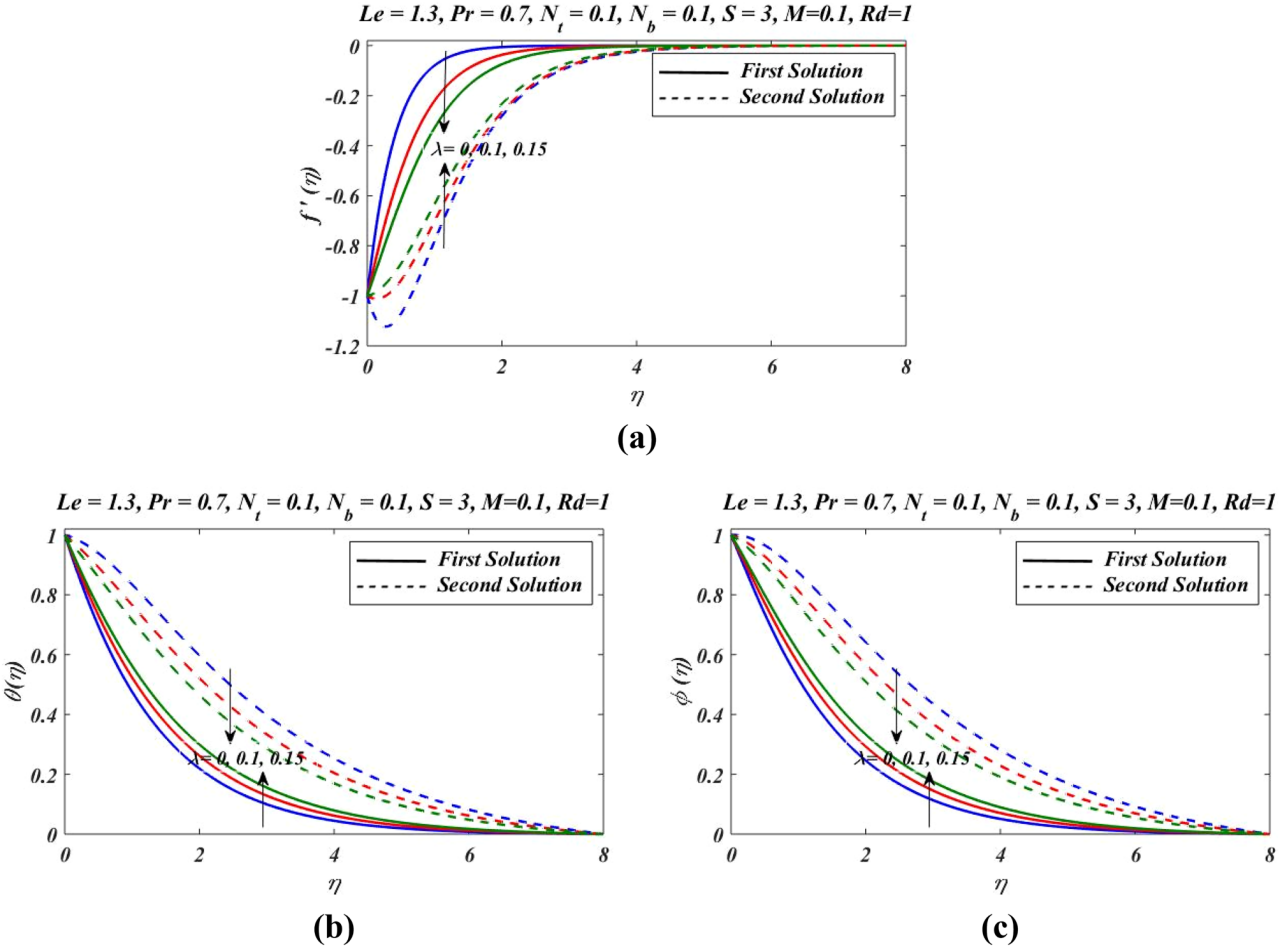
Outcomes of $\lambda$ on (**a**) velocity, (**b**) temperature and (**c**) nanoparticles volume fraction.

**Figure 4:**
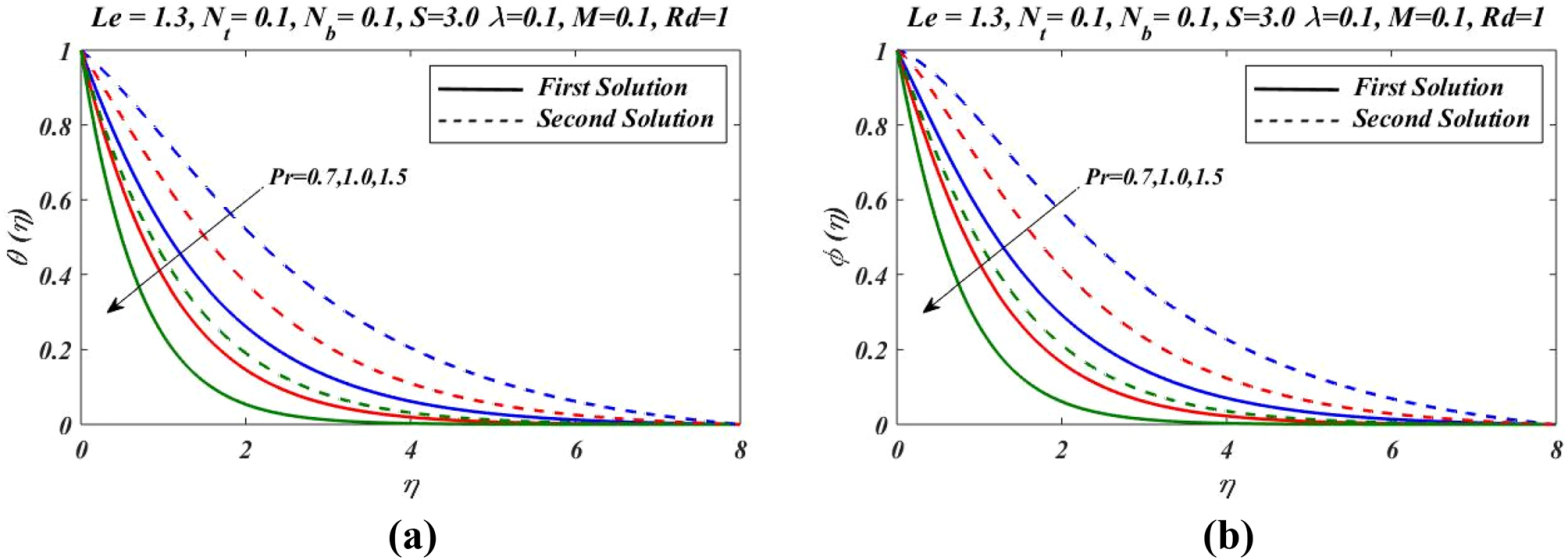
Outcomes of $Pr$ on (**a**) temperature and (**b**) nanoparticles volume fraction.

**Figure 5:**
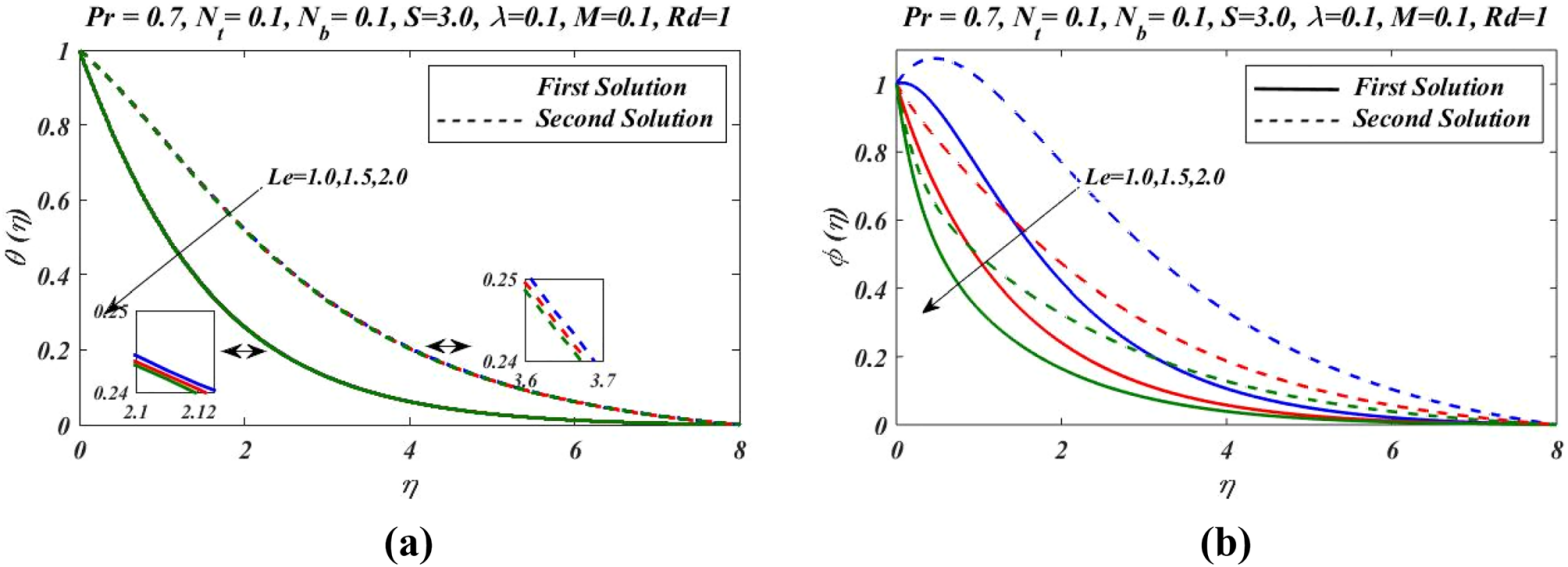
Outcomes of $Le$ on (**a**) temperature and (**b**) nanoparticles volume fraction.

**Figure 6:**
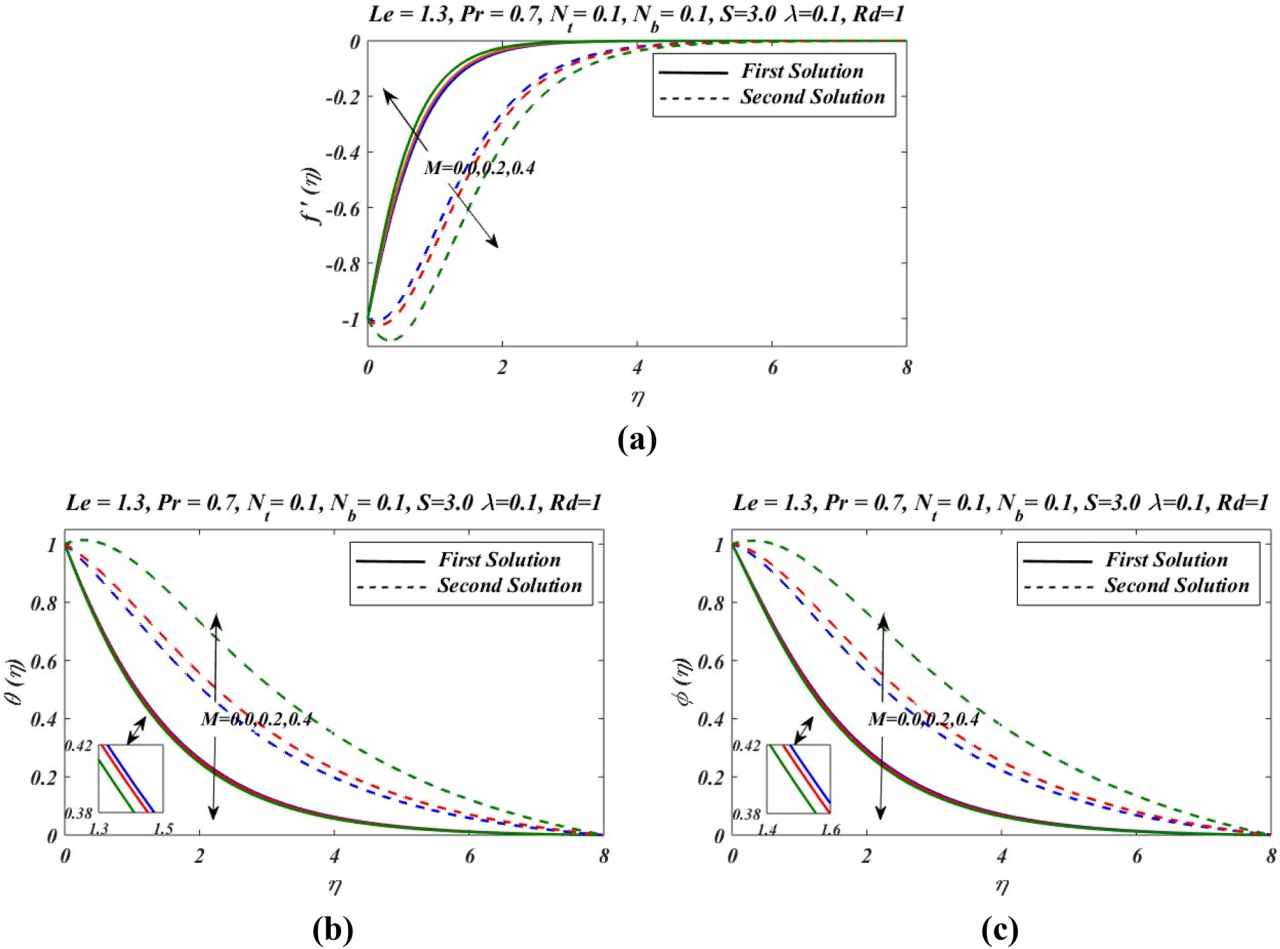
Outcomes of $M$ on (**a**) velocity, (**b**) temperature and (**c**) nanoparticles volume fraction.

**Figure 7:**
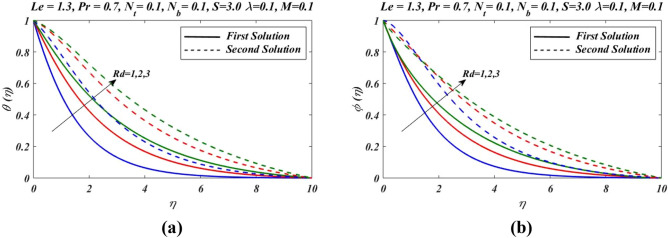
Outcomes of $Rd$ on (**a**) temperature and (**b**) nanoparticles volume fraction.

**Figure 8:**
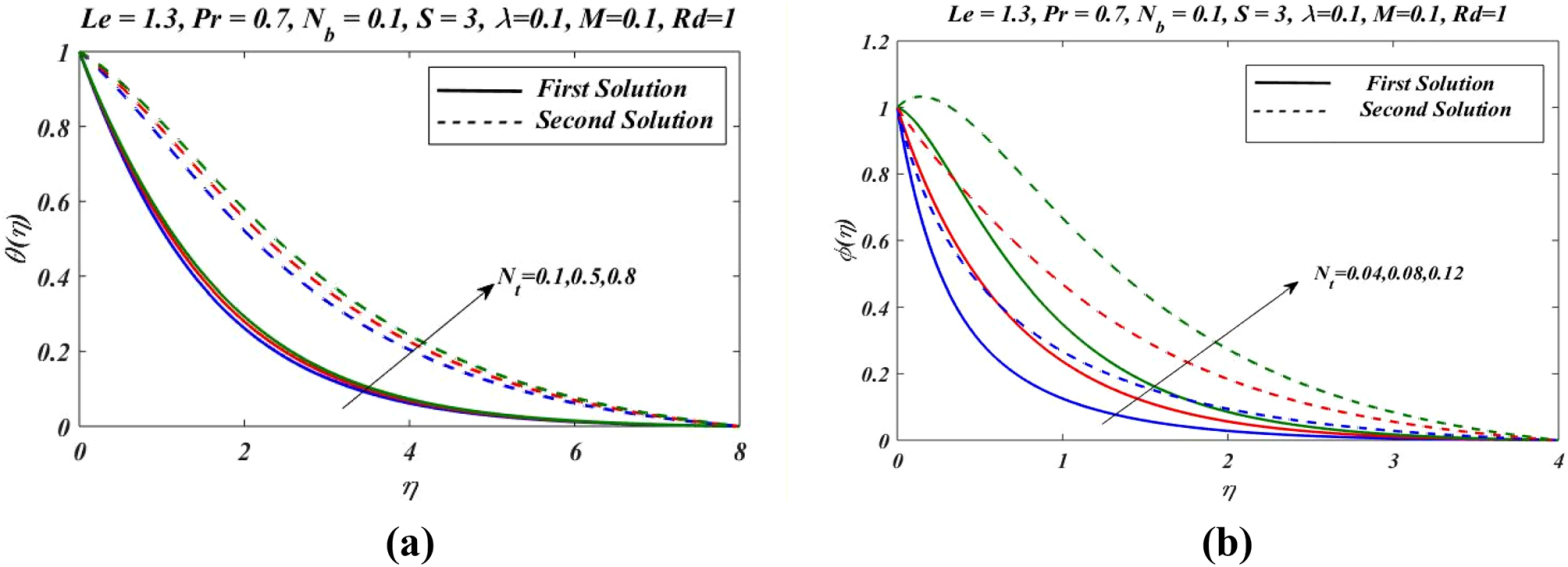
Outcomes of ${N}_{t}$ on (**a**) temperature and (**b**) nanoparticles volume fraction.

**Figure 9:**
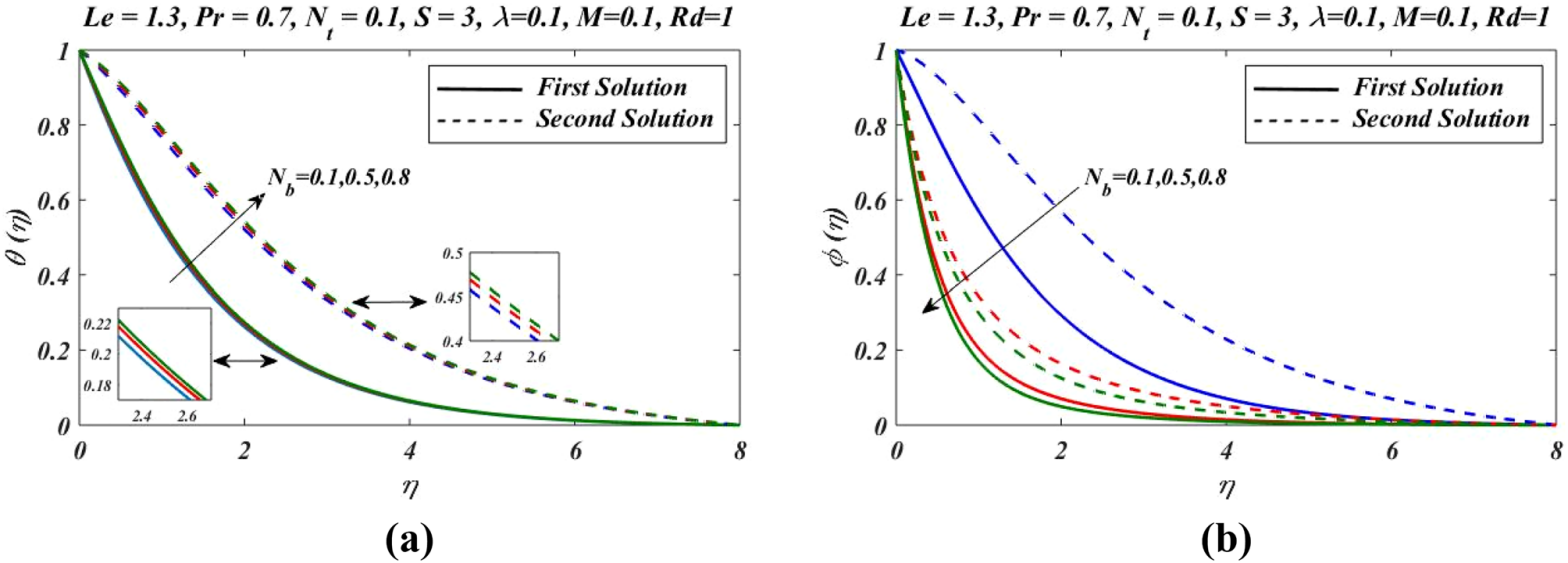
Outcomes of ${N}_{b}$ on (**a**) temperature and (**b**) nanoparticles volume fraction.

**Figure 10:**
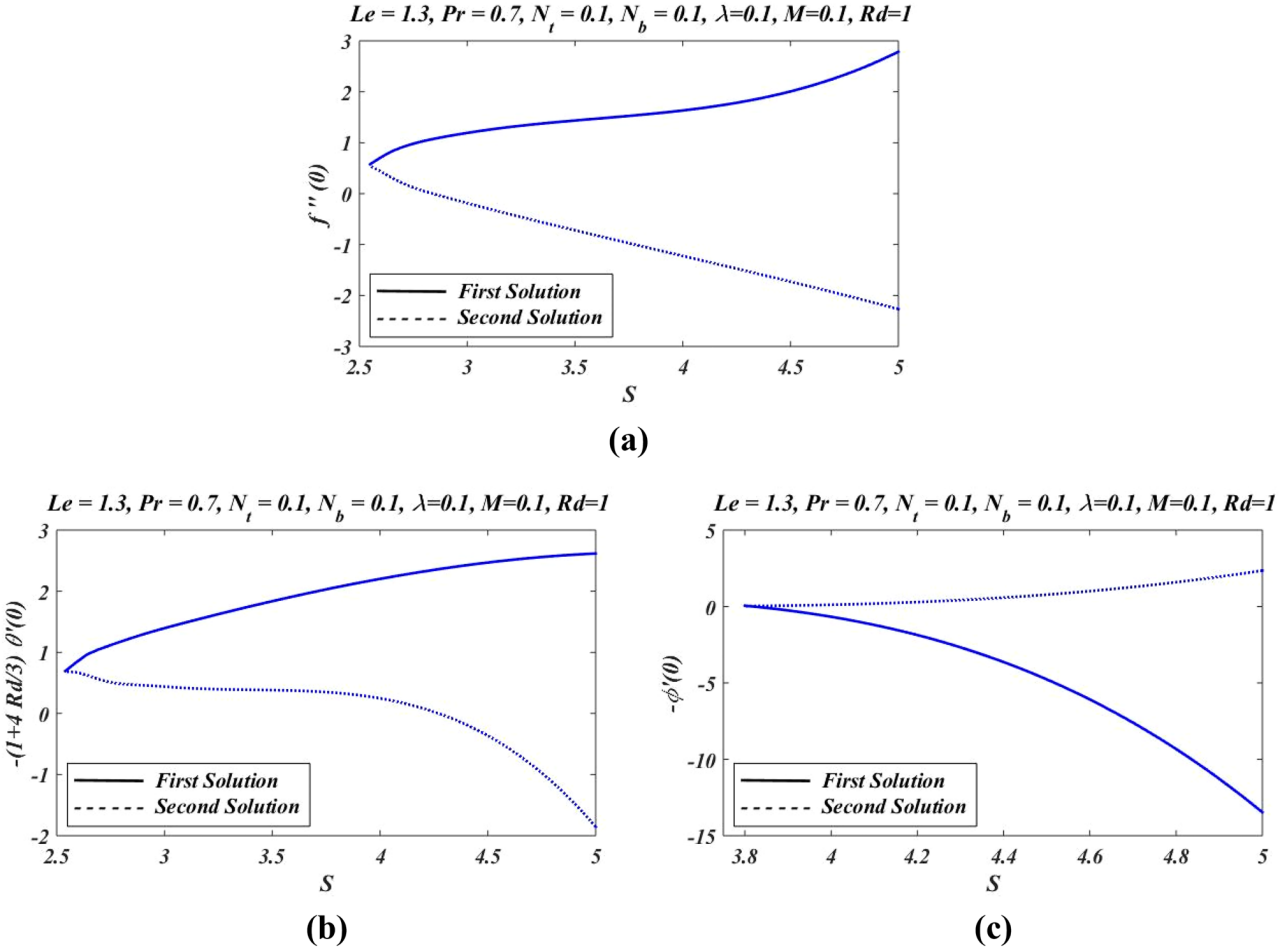
Graphs of (**a**) skin friction, (**b**) temperature gradient and (**c**) nanoparticle volume fraction profiles.

We will discuss the non-singular solutions for different values of participated parameters for ${f}^{\mathrm{^{\prime}}}\left(\eta \right), \theta \left(\eta \right)$ and $\phi (\eta )$ where ${f}^{\mathrm{^{\prime}}}\left(\eta \right)$ represents velocity profile, $\theta \left(\eta \right)$ shows temperature distribution and $\phi (\eta )$ shows concentration. Here, we will deal with the gradient of velocity at wall, temperature near wall and nanofluid concentration at surface wall for distinct variation of participated parameters. Our presented graphs given in Figs. 2, 3, 4, 5, 6, 7, 8, 9 and 10 will presents all these facts mentioned above.

Figure 2a–c presents the relative graph showing the variation of the suction/blowing parameter effects on different values of velocity ${f}^{\mathrm{^{\prime}}}(\eta )$, temperature $\theta \left(\eta \right)$ and concentration $\phi (\eta )$ fields. In Fig. 2a, it is noted that velocity ${f}^{\mathrm{^{\prime}}}(\eta )$ increases and decline in first and second zone of solutions, respectively. The observations regarding the nature of boundary layer revel that boundary layer is thinner and thicker in first and second branch respectively. Figure 3a–c signified the effects of suction/blowing parameter on temperature $\theta \left(\eta \right)$ and concentration $\phi (\eta )$, which shows identical behaviour. It reveals that when suction parameter $S$ is increased, $\theta \left(\eta \right)$ and $\phi (\eta )$ both reduces in both zones of solutions. Thus vorticity diffusion is confined when thickness of momentum boundary layer is decreased. This happens when suction fluid appeared to surface. Figure 3 illustrates the effects of different values of relaxation parameter $\lambda$ on different values of velocity, temperature and concentrationfields. In Fig. 3a, it is observed that by increasing relaxation parameter $\lambda$, the velocity ${f}^{\mathrm{^{\prime}}}(\eta )$ decreases and increases for first and second branch of solution respectively. Figure 3b indicates that by increasing relaxation parameter $\lambda$, temperature $\theta \left(\eta \right)$ profile increases and depressed in first zone and second branch of solution respectively. Similarly by increasing relaxation parameter $\lambda$, concentration $\phi (\eta )$ profile get increasing curve in first zone but declines in the second zone of solution. Figure 4a,b demonstrate effects of different values of Prandtl number $Pr$ on temperature $\theta \left(\eta \right)$ and concentration $\phi (\eta )$. These figures shows that with increase in $Pr$ temperature and concentration decreases remarkably. As $Pr$ is ratio of the viscous diffusion rate and thermal diffusion rate. Thermal diffusivity becomes weaker with the increase in $Pr$, consequently thermalboundary layer thickness dispirited in this phenomenon. It is remarked that nanoparticles volume fraction get slower when Prandtl number is increased. The outcomes for $\theta \left(\eta \right)$ and $\phi (\eta )$ due to Lewis number $Le$ are claimed in Fig. 5a,b. Figure 5a analysed that by increases in Lewis number $Le$, temperature $\theta \left(\eta \right)$ decreased for both branch solutions, in case of, nanoparticle volume fraction $\phi (\eta )$ decreases remarkably for both branches of solution. As Lewis number is the ratio between thermal diffusivity to mass diffusivity or it can be expressed as ratio between Prandtl and Smith number, so nanoparticles increases, as shown in Fig. 5b.This figure also shows a weaker nanoparticles concentration because of lower Brownian diffusion co-efficient by the increase in Lewis number. This is because Lewis number is associated with Brownian diffusion coefficient. Figure 6 demonstrate effects of different values of Hartmann number $M$ on different values of ${f}^{\mathrm{^{\prime}}}(\eta )$, $\theta \left(\eta \right)$ and $\phi \left(\eta \right)$. In Fig. 6a reveals that by increasing Hartmann number $M$, the velocity ${f}^{\mathrm{^{\prime}}}\left(\eta \right)$ increases and reduces in first and second zones, respectively. Figure 6b indicates that by increasing Hartmann number $M$, temperature $\theta \left(\eta \right)$ profile decreases and increases for first and second branch of solution respectively.

Similarly by increasing Hartmann number $M$, concentration $\phi (\eta )$ profile decreases in first zone of solution and attained at maximum level in second solution branch. Figure 7 demonstrate effects of different values of radiation parameters $Rd$ on $\theta \left(\eta \right)$ and $\phi \left(\eta \right)$. It is noted that by the increase in radiation parameter $Rd$, both temperature $\theta \left(\eta \right)$ and concentration $\phi \left(\eta \right)$ are increased.As we have ${N}_{t}$ thermophoresis parameter which is ratio of diffusion of nanoparticles to the thermal diffusion on nanofluids. The convenient of thermophoresis parameter ${N}_{t}$ on nanofluid temperature $\theta \left(\eta \right)$ and concentration $\phi \left(\eta \right)$ is proceeded in Fig. 8. The enhanced change in $\theta \left(\eta \right)$ and $\phi \left(\eta \right)$ is reflected with thermophoresis parameter (Fig. 8a,b). The thermophoretic force express the development of particles movement from heated to cooler zone which enhanced with ${N}_{t}$, and subsequently nanoparticles volume fraction increased and the temperature between fluid and the sheet is also increased as the result, thermal boundary layer is also increased.

Figure 9a illustrates effects of variation of Brownian motion parameter ${N}_{b}$ on temperature. It is noticed that increases in temperature for both solutions zones as Brownian parameter is increased. The graphical outcomes observed in Fig. 9b claim the impact of Brownian constant ${N}_{b}$ on $\phi (\eta )$. With the increases in Brownian motion parameter ${N}_{b}$ the thermal boundary layer thickness increased. But in case of nanoparticle volume fraction $\phi (\eta )$ we noticed opposite effects. With change in ${N}_{b}$, $\phi (\eta )$ decreases. Nanoparticles produces the Brownian motion. So Brownian motion is clearly effected by increasing ${N}_{b}$. For various values of suction parameters $S$, skin friction co-efficient, Nusselt number and Sherwood number are presented in Fig. 10. It gives clear picture of existence of dual solutions in all the graphs. For the first branch in Fig. 10a, with the increase in suction parameter S, skin friction co-efficient increases. Whereas for the second solution opposite nature of skin friction has been noticed. Similarly in Fig. 10b,c with the increase in suction parameter $S$, Nusselt number and Sherwood number increases in the first branch of solution whereas it is opposite for second branch of solution.

## Conclusions

In the presence of heat and mass fluxes, the steady boundary layer flow and heat transfer of Maxwell nanofluid with MHD and thermal radiation effects is studied over an exponentially contracting porous sheet. The foremost objectives of this investigation are presented below:

- As compared to linear shrinking sheet, exponentially shrinking sheet generates greater vorticity.
- With increase in suction parameter, diffusion of vorticity stops and transfer of heat from surface to fluid is increased.
- The decrease in nanoparticles volume fraction and temperature is noted with the increase in Lewis number.
- With the increase in Prandtl number, thermal boundary layer thickness, nanoparticles volume fraction and temperature are declined.
- Increasing Brownian motion parameter acts differently for temperature and nanoparticles volume fraction. Nanoparticles volume fraction decreases and temperature increases. Temperature at the wall also increases with the increase in Brownian motion parameter. With increase in thermophoresis parameter, nanoparticles volume fraction and temperature both increases. The nanofluid concentration and temperature get improved in this situation.
- With the increase in relaxation parameter, nanoparticles volume fraction and temperature distribution increases whereas velocity profile decreases.
- With the increase in Radiation parameter, nanoparticles volume fraction and temperature distribution increases.

## List of symbols

$u$: Velocity component in $x$ direction
$v$: Velocity component in $y$ direction
$$T$$: Temperature
${T}_{\infty }$: Constant free stream temperature
$$\upsilon $$: Kinematic viscosity
$({\rho c)}_{p}$: Effective heat capacity of nanoparticles
$({\rho c)}_{f}$: Heat capacity of the base fluid
$N$: Nanoparticle volume fraction
${D}_{B}$: Brownian diffusion coefficient
${D}_{T}$: Thermophoresis diffusion coefficients
$$U$$: Shrinking velocity
${q}_{W}\left(x\right)$: Variable surface heat flux
$${U}_{O}$$: Reference velocity
$${T}_{O}$$: Temperature flux
$${q}_{Wo}$$: Heat flux
$\frac{{\left(\rho c\right)}_{P}}{{\left(\rho c\right)}_{f}}$: Ratio of effective heat capacities of nanoparticle to nanofluid
${q}_{npo}$: Surface nanoparticle flux
$${N}_{o}$$: Nanoparticle fraction
$V\left(x\right)$: Velocity at the wall
$${v}_{O}$$: Constant
$B$: Variable magnetic field
$${B}_{O}$$: Constant
$$k$$: Thermal diffusivity
$$Pr$$: Prandtl number
$$Le$$: Lewis number
$$Rd$$: Radiation parameter
${q}_{np}\left(x\right)$: Variable surface nanoparticle flux

## Greek letters

$$\alpha $$: Thermal conductivity
$\beta$: Coefficient of thermal expansion
$$\sigma $$: Electrical conductivity
${\rho }_{f}$: Base fluid’s density
$${\Lambda }_{1}$$: Relaxation time
