# Author Correction: Dual solution framework for mixed convection flow of Maxwell nanofluid instigated by exponentially shrinking surface with thermal radiation

**DOI:** 10.1038/s41598-022-16239-7

**Published:** 2022-07-13

**Authors:** Qiu‑Hong Shi, Bilal Ahmed, Sohail Ahmad, Sami Ullah Khan, Kiran Sultan, M. Nauman Bashir, M. Ijaz Khan, Nehad Ali Shah, Jae Dong Chung

**Affiliations:** 1 Department of Mathematics, Huzhou University, Huzhou, 313000 People’s Republic of China; 2 Department of Mathematics and Statistics, The University of Lahore, Sargodha Campus, Sargodha, 40100 Pakistan; 3 Department of Mathematics, COMSATS University Islamabad, Sahiwal, 57000 Pakistan; 4 Department of Mathematics and Statistics, Riphah International University, I-14, Islamabad, 44000 Pakistan; 5 Nonlinear Analysis and Applied Mathematics (NAAM) Research Group, Department of Mathematics, Faculty of Science, King Abdulaziz University, P.O. Box 80257, Jeddah, 21589 Saudi Arabia; 6 Department of Mechanical Engineering, Sejong University, Seoul, 05006 Korea; 7 Department of Mathematics, Lahore Leads University, Lahore, Pakistan

Correction to: *Scientific Reports* 10.1038/s41598-021-95548-9, published online 05 August 2021

In the original version of this Article, Jae Dong Chung was incorrectly affiliated with ‘Department of Mathematics, Lahore Leads University, Lahore, Pakistan. The correct affiliation is listed below.

6. Department of Mechanical Engineering, Sejong University, Seoul 05006, Korea

The original Article has been corrected.

